# A hybrid scanning mode for fast scanning ion conductance microscopy (SICM) imaging

**DOI:** 10.1016/j.ultramic.2012.06.015

**Published:** 2012-10

**Authors:** Alex Zhukov, Owen Richards, Victor Ostanin, Yuri Korchev, David Klenerman

**Affiliations:** a The University of Cambridge, The University Chemical Laboratory, Lensfield Road, Cambridge CB2 1EW, UK; b Division of Medicine, Imperial College School of Medicine, Medical Research Council Clinical Sciences Centre, Hammersmith Campus, Du Cane Road, London W12 0NN, UK

**Keywords:** Fast scanning ion conductance microscopy, Live cell imaging, FPGA, A6 cells

## Abstract

We have developed a new method of controlling the pipette for scanning ion conductance microscopy to obtain high-resolution images faster. The method keeps the pipette close to the surface during a single line scan but does not follow the exact surface topography, which is calculated by using the ion current. Using an FPGA platform we demonstrate this new method on model test samples and then on live cells. This method will be particularly useful to follow changes occurring on relatively flat regions of the cell surface at high spatial and temporal resolutions.

## Highlights

► We introduce a new method for fast scanning ion conductance microscopy (FSCIM). ► Method based on adaptive scanning using a field programmable gate array. ► Method gives improved sampling rate compared to other SICM control methods. ► Comparison of hopping mode SICM and FSICM made on live A6 cells.

## 1 Introduction

Scanning ion conductance microscopy (SICM) is a form of scanning probe microscopy based on a nanopipette that uses the reduction in ion current as the pipette approaches a surface for distance feedback control [1]. SICM has largely been developed for non-contact live cell imaging. This poses specific imaging problems since the surface can have large abrupt changes in height and can also change with time due to cellular dynamics. It is important to develop new methods for distance feedback control that allow faster scanning as well as high resolution to follow topographic changes on the cell surface during important biological processes. Such methods, although developed for SICM, could also then be applied to other forms of scanning probe microscopy.

There have been several methods developed for scanning the nanopipette in SICM. The simplest method is DC mode scanning where the pipette is brought close to the surface, and raster scanned over the sample laterally, the *z*-position of the nanopipette is then adjusted vertically to maintain a constant current [2]. This mode of scanning is prone to probe crashing due to electrode current drift or a large fluctuation in sample height. This mode of scanning is now rarely used. In order to remove the problem of electrode drift the AC scanning mode was developed [3,4]. In this mode the pipette is modulated above the sample surface when scanning. This means the feedback signal is the gradient of ion current change and allows more stable imaging. The AC mode still has problems with large shifts on height of the sample. To address this issue the hopping mode has recently been developed for the reliable imaging of steep structures [5,6]. The hopping mode uses rapid descent from a safe *z*-position above the surface to a distance where the ion current is reduced to a certain value, allowing the position of the surface to be determined. The pipette is then withdrawn and the height of another position on the surface measured. This method is very stable, not sensitive to ion current variations and allows the scanning of very complicated surfaces with lots of high features. Adaptive scanning has been developed to deal with the issue that this mode is intrinsically slow due to the long time the probe spends away from the surface not determining its topography. This is done by recording lots of topographic information within places of interest and only briefly scanning other parts of the image. This step is crucial for scanning large areas with a high sampling rate, since otherwise it will take too much time to measure each point of the surface [6].

In this paper we describe another method of scanning which aims to gain as much information about the sample surface topography as possible during the same fixed time—fast SICM. Hopping mode will be the fastest method only for complex surfaces with very steep slopes when the pipette has to travel a lot along the *Z*-axis to follow the sample. But another situation is common, when there is a comparatively flat sample with features of the same scale as that of a pipette tip. The large pipette motions in hopping mode are not the best solution in this case as one cannot record surface topography when the probe is away from the surface and for this reason we have developed a fast SICM mode.

## 2 Materials and methods

### 2.1 Solutions

Phosphate buffer solution (PBS) was prepared by dissolving NaCl (Sigma) and sodium phosphate (Sigma) in deionised water measuring 18.2 MΩ resistance at 150 mM and 20 mM concentrations respectively. The pH of this solution was then adjusted to 7.2 using 1 M HCl (Sigma). Sodium chloride solution was made in the same manner as stated above but to concentrations of 150 mM and 3 M. All solutions were filtered to 20 nm using Anotop syringe filters (Whatman). Sodium bicarbonate (Sigma) solution was prepared as above to 7.5% concentration but without fine filtering.

### 2.2 Cell culture

#### 2.2.1 A6

A6 South African clawed toad kidney epithelial cells (ECACC) were cultured in flasks (Nunc) containing as media: in 500 ml 187.5 ml Leibovitz's L15 culture medium, 187.5 ml Ham's F12 medium, 50 ml Foetal Calf Serum (FCS), 10 ml L-Glutamine, 10 ml Penn Strep ([+] 10,000 units/ml Penicillin, [+] 10,000 μg/ml Streptomycin; all GIBCO) and 1.5 ml 7.5% sodium bicarbonate solution and then made up to 500 ml total volume using deionised water, approximately 50 ml [7]. The solution was then filtered to 200 nm. Imaging experiments were carried out between 15 and 25 passages. Cells were maintained at 25 °C in an atmosphere of humidified air in a passive desktop incubator (home built). Cells were seeded on to 35 mm dishes (Nunc) for imaging, having been split from the bulk flask using 0.25% Trypsin–EDTA 0.05% (GIBCO). The cells were left for 1–4 days to reach 95% confluence before being imaged.

#### 2.2.2 Cos7

Cos7 Cells were cultured in a flask containing as medium: 440 ml Dulbecco's Modified Eagles Medium (DMEM), 50 ml FCS, 5 ml L-Glutamine and 5 ml Penn Strep (all GIBCO). Cells were maintained at 37 °C in a humidified atmosphere supplemented with 5% CO_2_. Cells were split with trypsin from the flask and seeded onto 35 mm dishes (Nunc) for imaging.

### 2.3 Instrumentation

**Fig. 1:**
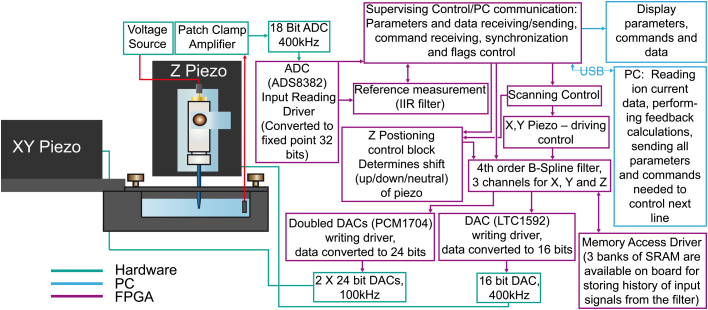
Schematic of FSCIM instrument showing how the FPGA interacts with the hardware components.

The SICM, as used for the hopping mode experiments, has been described in detail previously [3,6,8,9]. Modifications to this setup, for the fast SICM experiments, are outlined below. For better performance and lower cost it was decided to base the control system on a field programmable gated array (FPGA) as used previously by Takahashi et al. [10]. Since fast prototyping was important, an FPGA-based RC-10 board (Mentor Graphics; recently replaced by the RC-240 board) along with the development kit and high-level programming language Handel-C (Mentor Graphics) was used. The resulting design had some self-built and some evaluation boards connected to the FPGA board via a ribbon cable and a PC communicating with the FPGA via a USB interface. A fast 18-bit 600 kHz ADC (ADS8382) was used for ion current readings, an accurate and fast 16-bit DAC LTC1592 controlled the *Z*-position of the piezo-stage, and two slower and more accurate DACs PCM1704 24-bit (running at 100 kHz) controlled the *XY* positioning. A ready to use solution from Physik Instruments combining a multi-channel amplifier (E-509.C3A) and piezo-actuators (25 μm P-753.21C for *Z* and 100 μm P-621.2CL for *XY*) was used at the output. Every input and output operation of the control is driven by the FPGA, but a more complex analysis was run on the PC. The FPGA controls the scanning of one line, reading of the ion current reference signal, and the sending of ion current readings to the PC. The PC stores the results for ion current and the pipette *z*-position along the line and then prepares a new set of heights for the next line to be scanned by the FPGA. Switching to a bigger FPGA chip with significant external memory installed on the board allowed the addition of digital filtering to the FPGA, where by all outputs pass through a cubic B-spline Finite Impulse Response (FIR) filter to accurately damp spurious mechanical oscillations at resonance frequencies. The PC still remained a major component, controlling the distance feedback (although no rapid decision making is needed during the scanning of one line, so the PC performance was sufficient) and all data interpretation. The instrument is shown in schematic form (Fig. 1).

### 2.4 Pipettes

Pipettes were pulled using a P-2000 CO_2_ laser puller (Sutter) from 1 mm external diameter, 0.50 mm internal diameter glass capillaries (Sutter). When fabricated the pipettes had a tip aperture of approximately 50 nm diameter, allowing resolution between 25–150 nm per pixel [11].

### 2.5 Samples

A UV diffraction grating with 3600 grooves per mm was purchased from Edmund Optics. An AFM height calibration standard was purchased from Agar Scientific (model number HS-1400 MG). The standard had features with a step height of 113 nm. Both samples were glued into a 35 mm (Nunc) dish for imaging.

## 3 FPGA-based realization

### 3.1 Principles of method

**Fig. 2:**
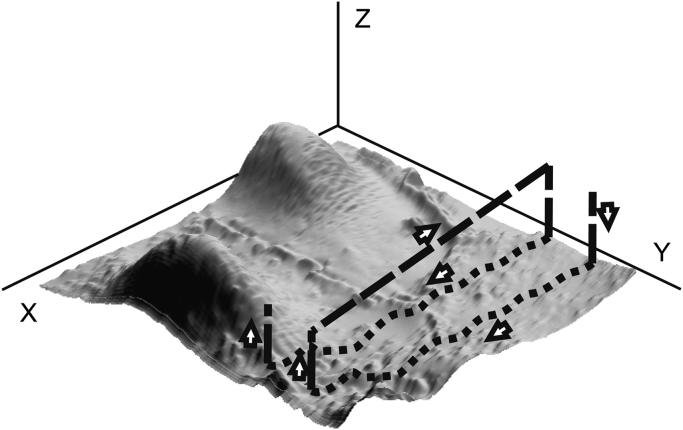
FSICM scan cycle. The scanning starts from the right hand corner. After measurement of the ion current reference, a few points of the first line are acquired in the hopping mode to produce a rough line image; this is then corrected with a few iterations in the fast scanning regime (5–10) to give an accurate first line. When the line is complete the pipette is withdrawn to a safe distance and moved back to the start of the next line as shown; this is then recorded using the first line as a reference. The image shows a live Cos7 cell. This is a 90×90 μm^2^ area scanned using 600 lines and took about 12 min to acquire a total of 614,440 pixels.

**Fig. 3:**
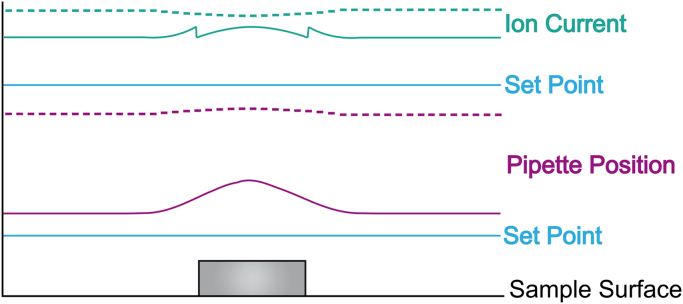
Modelled example of the distance control. The rectangular feature on the sample surface has relatively sharp edges. The pink solid and dotted curves show how the control will drive the pipette at close (large) and far (small) current set points respectively. The corresponding ion currents are shown as solid and dotted green lines. (For interpretation of the references to colour in this figure, the reader is referred to the web version of this article.)

The feedback control for the proposed method is a bit unusual. Instead of trying to control the *z*-position of the pipette while it is doing a scan of one line, which would profoundly slow down the rate, all feedback calculations are done when the scanning of the line is over. The PC makes a correction to the path so that next time the line is being scanned (or the next line if it uses the current one as a prediction) a new corrected path will be used (Fig. 2). The method is strongly based on the idea of image reconstruction and an assumption that the driving path is not necessarily coinciding with the true profile of the line. So the concept of a set point for feedback is slightly modified. Now the control has two different current set points: one is for upper limit of ion current and another for lower. Before starting the scan of each line the infinity ion current reference is measured away from the sample (*I*_max_). Reaching the upper current limit during the line scan at a certain lateral position indicates that the path information around that place must be modified so that all further scans of the line will go closer to the surface there; it gives non-zero error function of one sign. The reverse action is applied when the current goes lower than the second set point value as that means the detected distance from sample was not safe for crash free scanning. When either of those events happens on a particular point of a scanned line its path is supposed to be adjusted. It is clear that the actuation control system can reproduce only low spatial frequency features, so to reproduce high spatial frequency features a scaled convolution of error function and a fixed width Gaussian function are used to calculate the correct topography. Fig. 3 shows a modelled example of how the control will drive the pipette along a surface with a sharp-edged feature. Two different distances from the surface are shown. It is obvious that none of the pipette position traces are suitable for producing an accurate surface topography on their own, while both the pipette position and ion current might be used to recalculate the profile accurately. This also explains why in real scans where the recorded ion current has noise those paths, which are closer to the surface, will produce more accurate and less noisy reconstructions, as the signal to noise ratio will be better and the curve used to correct the error will also be more accurate. The *XY*-scanning is performed along parallel lines—raster scanning. Not having a defined current set point makes it possible to scan in a quasi-constant-height mode, similar to that of AFM but still following the overall coarse changes in surface topography. Therefore the nearest analogue to fast SICM is the error signal mode in AFM where the error signal is used essentially as a high pass filter to help resolve features with a higher spatial frequency [12].

**Fig. 4:**
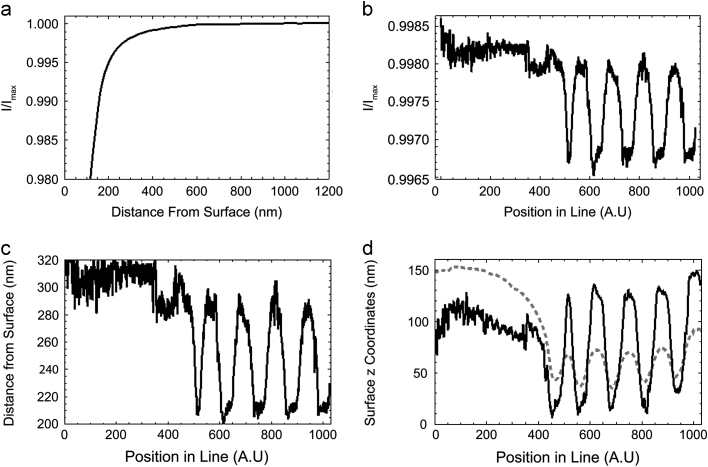
(a) Approach curve, dependence of ion current versus distance to the surface, (b) ion current recorded for one line of a scan, (c) recalculated distance between pipette tip and surface along the scanned line using the method referred to in Section 3.1., and (d) the dashed grey line shows the pipette tip trajectory (shifted down for easier comparison) and the solid black line is the result of the reconstruction of the surface shape. In this example the sample is an AFM calibration standard with a known height of 113 nm. It is clear from the position of the pipette (c) during the line scan, that it does not image the surface accurately but the reconstructed curve (d) correctly determines the true height.

Reconstruction of the surface topography is now a very important part of image processing. Fig. 4 shows data processing for one scanned line. We use the trajectory of the pipette tip and the pipette approach curve to show how the ion current depends on the distance from the surface. The approach curve data can be used to calculate the clearance of the pipette from the surface as a function of ion current reduction. This additional height can then be subtracted from the original pipette trajectory, using the measured ion current reduction, to improve the reconstructed profile and make it closer to the real one.

**Fig. 5:**
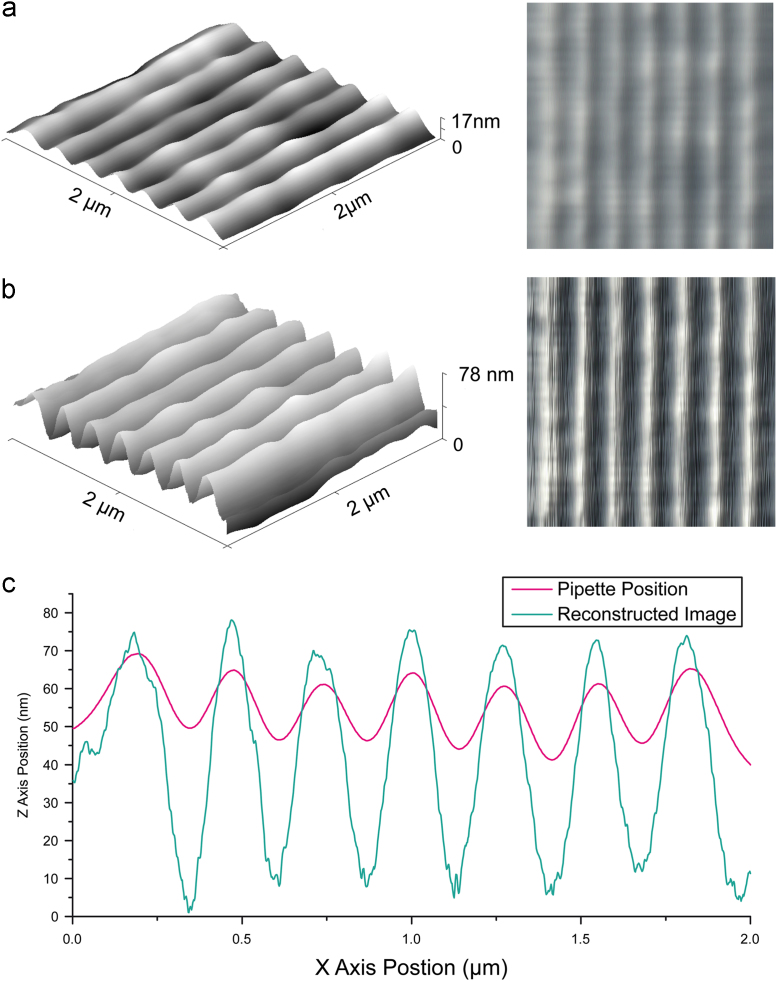
Scan of a UV diffraction grating (3600 grooves/mm) under PBS buffer. Image (a) is obtained from the pipette position and image (b) was reconstructed using the pipette positional data, the ion current data and the approach curve. The two pictures on the right have the same height greyscale. Note the difference in contrast and position of the grooves. The height in the left picture is 17 nm, and for the right one it is 78 nm. The image was scanned in 50 lines at 102.4 ms per line and smoothed with a 1 point mean value filter, the image was recorded in 7 s. (c) Graph of a single line each of (a) and (b); note the slight shift in the peak centres of the pipette position versus the reconstructed image.

In order to make the correction to the scanned surface profile a calculation based around a pipette approach curve is used. It is important that this approach curve is acquired before or just after scanning the surface and for each particular pipette. To negate the effects of bringing the pipette to a highly sloped surface, which would affect the shape of the approach curve or worse, break the pipette, only relatively flat regions are chosen for the procedure. To record the approach curve the PC software curtain steps the pipette closer to the surface in discrete steps, usually between 1 and 5 nm, while recording an average ion current value for each position of the *z*-piezo until a 5% reduction in ion current is achieved. This means an approach curve containing few features is obtained and can be approximated by a polynomial function. Another approximation works well and was used for all reconstruction performed in this paper; it is as follows: *f *(*x*)=*α*(*x*−1)+*β*(*x*−1)2+*γ *Arctan(*δ*(*x*−1)), where *x* is an independent variable equal to *I/I*_max_ and *α*, *β*, *γ*, and *δ* are to be found according to the method of least squares. As the image will not change by adding any constant offset to the *z*-position we do not control the absolute distance to the surface by doing approaches, only finding out a relative coordinate for every ion current point. So the procedure of producing the image is as follows. Each pixel of data collected has a precise topographic *X*, *Y* coordinate an ion current and a non-topographically accurate *Z*-position. By taking the ion current from each point and feeding this into the approach curve we can find out the corresponding height; this is then added to or subtracted from the *Z* coordinate for each pixel; addition or subtraction depends on the sign of the approach curve being used. This then gives the accurate topography shown in Fig. 5b.

## 4 Results and discussion

The control system was tested on various samples. One of the simplest was a UV diffraction grating. The results are shown in Fig. 5. This picture is a good demonstration of how the original *z*-position map that was driving the pipette must be corrected with the ion current data. Not only has the scale of features changed, but their locations too. This was due to a slight drift in the piezo-actuators towards the left side of the image, causing the topographic information used for control of the next line scanned to be slightly incorrect. This incorrect spatial information used to drive the pipette caused deviations in the ion current, which when corrected by the procedure above, translated into changes in the *Z*-position and altered contrast in the processed image.

**Fig. 6:**
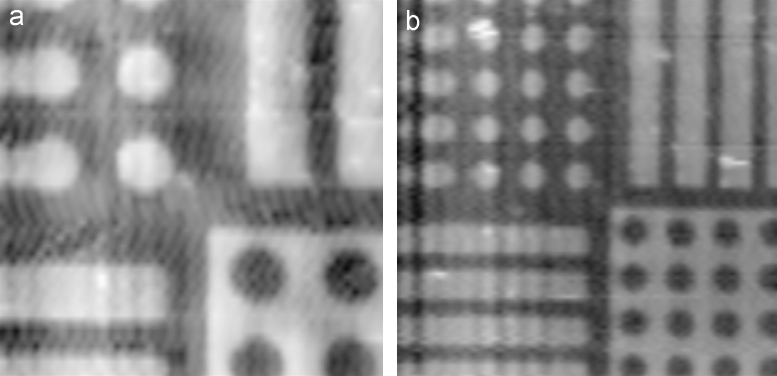
Images of an AFM calibration standard. (a) 20×20 μm^2^ scanned in 50 lines of 1024 pixels at 102.4 ms/line and (b) 40×40 μm^2^ scanned in 100 lines of 1024 pixels at 102.4 ms/line.

Another sample that was used to test the system was an AFM calibration standard. It had a set of lines, square and round pillars, and pits with known heights/depth. The goal was to achieve as fast a speed of scanning as possible. Fig. 6a shows a 20×20 μm^2^ scan of this standard. It took about 10 s to acquire the image. The imaging rate is limited by the data acquisition rate (that is 400 kHz in our case due to the fast FPGA and ADC), non-zero pipette capacitance and ion current amplifier filters. The next example in Fig. 6b is a larger scan of the same sample. The area is 4 times bigger but the scanning time has only increased by a factor of 2 (about 20 s); since the time to scan a line is always constant, due to the very flat topography making the line scan time negligible, only the number of lines scanned affects the total scan time. This also had an insignificant effect on the resolution. The scanning control was sufficiently stable that there was no crash between the pipette and the hard silicon/silicon oxide sample surface. Compared to comparative scan speeds with modulation mode SICM, repeated scanning of the same area of the sample was also far more stable and crashes of the pipette very rare.

**Fig. 7:**
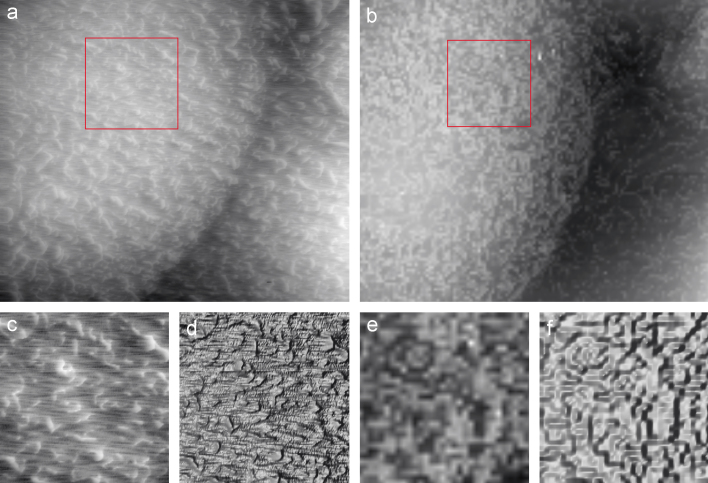
Comparison of images of A6 Cells taken with (a) Fast SICM, 600 lines of 1024 pixels at 409.6 ms/line completed in around 10 min, (b) hopping mode SICM 128×128 pixels taken in around 25 min both images 40×40 μm^2^. Panels (c) and (e) show normal grey scale zooms of the areas highlighted in (a) and (b) respectively and (f) and (d) show the same areas with a lighting effect added to highlight small features which otherwise might not be visible.

Fig. 7a shows the result of scanning live A6 cells. It took about 10 min to record the topography. The same sample scanned in the hopping mode with another DSP-based control system is shown in Fig. 7b and took around 25 min to record the topography. All amplifiers, piezos and mechanical system, and scanning pipettes remained the same and so it is easy to compare the results. The number of points sampled on the surface in fast SICM is significantly higher, providing more surface detail and the scan time was also shorter. However, this does not prevent observation of the microvilli on the cell surface, which have the same width and similar heights in both images, approximately 300 nm wide and 350 nm high for fast SICM and around 350 nm wide and 400 nm high for the hopping mode. The relative similarity between the widths and heights is due to the way in which the images are taken. The image in hopping mode is likely to disturb the microvilli's positions less but will capture their topography with far fewer pixels, leading to the artefacts observed in the zooms shown in Figs. 7e) and f). In contrast the rapid *X*–*Y* motion and smaller *Z*-axis motion of fast SICM is likely to bend the microvilli to the side slightly when it images them, causing some image blur offset by the large number of pixels used to capture the surface topography. Due to the dynamic nature of the microvilli on the cell surface [7,13] there is no continuity in their positioning on the cell surface between images taken with the hopping mode and fast SICM. Due to the huge number of points being scanned it is possible to resolve features in fast SICM that are not possible to resolve in the hopping mode in a reasonable time scale for a dynamic, live cell surface. A hopping mode image obtained to the highest resolution possible with the hopping mode software, 512×512 pixels would take around 5 h to complete compared to around 10 min for the 1024×600 pixel image in fast SICM.

**Fig. 8:**
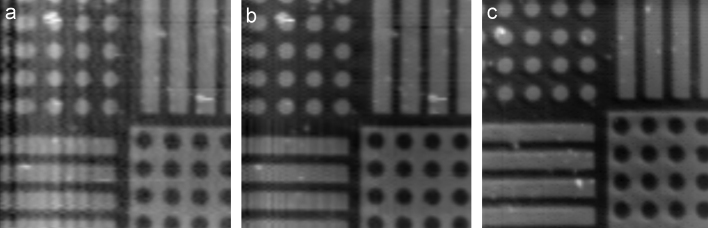
AFM calibration standard, shown at 3 different scan speeds: (a) 102.4 ms, (b) 204.8 ms and (c) 819.2 ms per line. Each scan contains 100 lines of 1024 pixels and is 40×40 μm^2^.

Fig. 8 shows three scans of the same sample taken at three different scan speeds. It is clear from looking at the images that the faster the image is recorded the lower the quality of the image obtained. The higher imaging speed will also add artefacts to the images due to the vibration of the scanning stage and the pipette when the stage is moved at high speed.

## 5 Conclusions

The new scanning mode introduced above works as we would expect. It gives a big rate boost in case of surfaces containing lots of small details with a size comparable with the scanning pipette size and even smaller heights. There is a trade-off between speed and quality of the image; on average, quality tends to be worse than that for the hopping mode, but the scanning speed is better. This is because while fast SICM can guarantee high scanning speed, it cannot guarantee quality (low noise and high resolution) since some features will be measured with the pipette at a large distance away from the surface. In contrast the hopping mode with adaptive scanning [6] that we used to compare our results with, cannot guarantee small features being skipped during the scanning because of their location and relatively flat surface around although each point is measured with the pipette at the same distance from the surface. Fast SICM also has the advantage of being able to collect more pixels per image than was possible or practical with the hopping mode, 1024 per line compared to a maximum 512×512 for the hopping mode. There is another big difference in that images can be post-processed to produce accurate topographies. In this sense our method is complementary to the hopping mode: it fast scans all points on the relatively flat surfaces whereas the hopping mode can slowly scan highly convoluted surfaces and find areas of interest for fast scanning. The mode is different from the DC mode, in which topographies like the one shown in Fig. 6a would not be obtainable in the same short time scales, as precise reproduction of every feature would take the control software much longer. Also the use of previous line estimation allows high speed scanning without frequent probe sample crashes. Currently the technique collects pixels at nearly 60 times the rate of the hopping mode SICM (depending on the speed at which the line is scanned). Potentially removing the PC from the control chain and moving its duties to the FPGA could double this speed increase. Currently the FPGA has to wait for the PC to prepare the scanning parameters for the next line scan; this often takes around the same amount of time as that of the line scan itself.

In conclusion fast SCIM provides a large increase in the imaging rate and number of points imaged for surfaces containing a lot of small details, with a size comparable or smaller than the scanning pipette size and will allow faster imaging of biological surfaces before they undergo dynamic changes in structure. This is particularly advantageous for small scans since for large scans drifts in the ion current during a single line are more significant. It therefore nicely complements the hopping mode, which is most advantageous for surfaces with high topography and can be used for large survey scans of samples. Since both modes use the same hardware they can be combined and fast SCIM can then be used for small rapid scans of relatively flat regions to obtain images at higher resolution and follow surface dynamics.

## Contributions

Alex Zhukov: method concept, control algorithm, FPGA programing, experiments and paper writing.
Owen Richards: experiments, paper writing and figures.
Victor Ostanin: control algorithm and electronics design.
Yuri Korchev: experimental design.
David Klenerman: experimental design and paper writing.
